# Anti-hypertensive medication access and affordability and their association with blood pressure control at a teaching hospital in Ghana

**DOI:** 10.11604/pamj.2021.39.184.27977

**Published:** 2021-07-08

**Authors:** Mark Amankwa Harrison, Afia Frimpomaa Asare Marfo, Mercy Naa Aduele Opare-Addo, Daniel Nii Amoo Ankrah, Franklin Acheampong, Frempomaa Nelson, Kwame Ohene Buabeng

**Affiliations:** 1 Pharmacy Department, Korle Bu Teaching Hospital, Accra, Ghana,; 2 Department of Pharmacy Practice, Faculty of Pharmacy and Pharmaceutical Sciences, College of Health Sciences, Kwame Nkrumah University of Science and Technology, Kumasi, Ghana

**Keywords:** Affordability, anti-hypertensive, blood pressure, hypertension, health insurance

## Abstract

**Introduction:**

many hypertensive patients require two or more anti-hypertensive drugs, but in low- and middle-income countries there may be challenges with medication access or affordability. The objective of this study was to determine accessibility and affordability of anti-hypertensive medicines and their association with blood pressure (BP) control among hypertensive patients attending the Korle-Bu teaching hospital (KBTH) polyclinic.

**Methods:**

a cross-sectional study was conducted among 310 systematically sampled hypertensive patients attending the KBTH Polyclinic in Ghana. A structured questionnaire was used to obtain data on patient demographics and clinical characteristics, prices, availability and mode of payment of generic anti-hypertensive medicines.

**Results:**

fifty-nine patients (19.4%) made out-of-pocket payments. At the private pharmacy and hospital, 123 (40.5%) and 77 patients (25.3%) respectively could not afford four anti-hypertensive medicines. Medicines availability at KBTH was 60%. Continuous access to BP drugs at KBTH was 14.8%. Overall access was 74.9% (SD ± 41.3). Out-of-pocket affordability of the medicines was positively correlated with BP control (R=0.12, p=0.037). Obtaining medicines via health insurance only was more likely to result in BP control than making any out-of-pocket payments (OR= 2.185; 95% CI, 1.215 - 3.927). Access at KBTH was more likely to result in BP control (OR=1.642; 95% C.I, 0.843 - 3.201).

**Conclusion:**

there were access challenges although most patients obtained BP medication free. Out-of-pocket affordability is a challenge for some hypertensive patients. Access to affordable BP medication can improve BP control. These findings provide an impetus for urgently evaluating access to affordable anti-hypertensive medicines in other hospitals in Ghana.

## Introduction

Globally, hypertension which is a significant cardiovascular disease risk affects over one billion people [1]. About 80% of cardiovascular-related deaths occur in low and middle-income countries [2,3]. In Ghana, hypertension is a public health problem with a crude prevalence range of 19-48% [4]. Although various classes of anti-hypertensive medicines are available in practice, blood pressure (BP) control remains poor in most developing countries, including Ghana [5-7]. Globally, only 32.5% of hypertensive patients have controlled blood pressure [5]. The control of blood pressure usually requires two or more blood pressure lowering medicines [8]. However, in many low- and middle-income countries, access to medicines is a challenge [9,10]. Where medicines are available, they are often not affordable, and where they are affordable, they may not be available [9]. While medication adherence among hypertensive patients may be a challenge [11,12], this may be accounted for, by affordability and accessibility challenges. In Ghana, the public sector supply chain is often inadequate to meet the medicine needs of health facilities, and health facilities procure largely from private suppliers [13]. The large proportion of imported medicines in Ghana often affects their pricing. The National Health Insurance Scheme (NHIS) was introduced by the government of Ghana to make medicines more affordable and improve access. However, patients enrolled on the health insurance scheme may make out-of-pocket payments when medicines are unavailable [14]. In facilities where medicines are supplied free, availability is the main challenge, hence out-of-pocket payments may still be made. In resource-limited countries such as Ghana, access to affordable anti-hypertensive medicines may not only be a challenge but is also likely to have an important influence on blood pressure control. Literature on access and affordability of anti-hypertensive medicines and their association with blood pressure control, particularly in Ghana is sparse. The objective of this study was to determine the affordability and accessibility of anti-hypertensive medicines and their association with blood pressure control in hypertensive patients. This study is expected to contribute to the development of health policy interventions targeted at improving trends in antihypertensive use, availability and access to anti-hypertensive medicines.

## Methods

**Study design and setting:** this was a cross-sectional study conducted among hypertensive patients attending the polyclinic department at the Korle Bu teaching hospital (KBTH). The Korle Bu teaching hospital is Ghana´s largest referral hospital and Africa´s 3^rd^ largest hospital with a bed capacity of 2,000. It has 17 clinical and diagnostic Departments/Units, and an average daily out-patient attendance of 1,500 and 250 in-patient admissions. The Polyclinic is one of the clinical departments, that has an average out-patient attendance of 59,000 annually. Hypertension is the leading cause of out-patient attendance with hypertensive patient attendance of 1300 in a month, and 14,549 in year 2017.

### Inclusion and exclusion criteria

**Inclusion:** hypertensive patients attending the polyclinic who had received pharmacotherapy for at least two months prior to the study were included.

**Exclusion criteria:** hypertensive patients attending the polyclinic who were less than 18 years of age; those on admission and those with stroke were excluded from the study.

**Sample size and sampling:** three hundred and ten (310) patients diagnosed with hypertension were enrolled into the study between January and February 2019. A sample size of 296 was obtained using the formula for calculating sample size, developed by the Open source statistics for public health for finite populations [15]. Systematic probability sampling was used to enroll every 3^rd^ hypertensive patient who met the inclusion criteria. Medical folders of hypertensive patients who had attended the polyclinic on clinic days were identified at the out-patient department (OPD), and those who met the inclusion criteria were identified for sampling. The respective patients and their contacts were listed. The investigators then randomly selected between the first three patients on the list as the starting point for sampling. From this random starting point, the investigators then selected every third patient from the list until the end of the list was reached. Patients identified were contacted by the investigator at the OPD for their consent to participate.

**Data collection:** a structured questionnaire was administered by trained research assistants (pharmacists) from the department of medicine of Korle Bu teaching hospital. Prior to data collection, the questionnaire was pretested and validated. The key variables investigated were affordability, access (accessibility) and blood pressure. Data collectors identified and interviewed hypertensive patients who were part of the inclusion criteria. Systematic sampling technique was used to include every 3^rd^ hypertensive patient. Patients were recruited after consenting to participate. Consent was obtained by patient reading, understanding, filling and signing the consent form after data collectors had explained the study to the patient. Information about each study participant´s demographic (age, sex, income, food expenditure, education level, health insurance subscription status, smoking, alcohol consumption) and clinical characteristics were collected. Each participant´s blood pressure in a sitting posture was measured twice after a 5 minute rest period, with the use of a validated digital sphygmomanometer. The data collectors ensured that BP measurements were done when the patient was in a relaxed posture, with the person quiet and seated, with their arm outstretched and supported. Data on anti-hypertensive drugs used by participants were obtained from the patients´ prescriptions. Information on study participants total monthly income and expenditure on food were also collected to assess their capacity to pay for BP lowering medicines. Data on the availability and retail price of six lowest priced generic anti-hypertensive medicine classes (including the 5 major classes) at the hospital pharmacy was collected. Price data on the drugs was also collected from 2 selected community pharmacies within the hospital´s catchment area using the WHO/HAI medicine price data collection form. Price and availability data was collected on standard doses of ten commonly prescribed anti-hypertensive drugs (Table 1).

**Table 1:** standard doses and quantities of anti-hypertensive medicines for estimating affordability for hypertensive patients (N=310) recruited from January to February 2019 at the Korle Bu teaching hospital (Ghana)

Medicine's name, strength and dosage form	No. of units for affordability analysis 30-day supply
Amlodipine 5mg tab	30
Nifedipine 20mg tab	60
Lisinopril 10mg tab	30
Losartan 50mg tab	30
Atenolol 50mg tab	30
Carvedilol 12.5mg tab	30
Bendroflumethiazide 2.5mg tab	30
Furosemide 40mg tab	30
Spironolactone 25mg tab	30
Methyldopa 250mg	120

**Data analysis:** data were analysed using statistical package for social sciences, SPSS version 22. Descriptive and inferential statistics were used. Categorical variables were expressed as frequencies and percentages, while continuous variables were described using means and standard deviation. Logistic regression analysis was used to determine the predictors of blood pressure control. Chi-square tests were utilized to determine the association between variables. Confounding factors for the relationship between variables were also tested. P value < 0.05 was considered statistically significant.

### Main outcome measures

**Blood pressure control:** was defined as BP<140/90mmHg for all patients based on the 2018 European Society of Cardiology guidelines which sets an initial treatment target of 140/90mg for all patients [16]. Blood pressure control was defined as an average BP of less than 140/90mmHg from two measurements.

**Anti-hypertensive medicine costs:** were determined using the monthly supply cost of the standard dose of the lowest priced generic;

**Patient´s capacity to pay:** was defined as total monthly income minus expenditure on food.

**Affordability:** was determined based on the ability to purchase anti-hypertensive medicines. The medicines were considered affordable if the total monthly retail cost of the lowest priced generic anti-hypertensive medicines was less than 20% of patients´ monthly capacity to pay, in line with the literature on catastrophic health expenditure [17].

**Accessibility:** was measured as access at the hospital and overall access.

**Access at the hospital:** was measured as Availability of medicines at the hospital pharmacy and the proportion of patients who could always obtain them at the pharmacy. The availability target was 80% (WHO target). Access at the hospital was rated as very low (<50%), low to medium (50% - 80%), medium to high (81% - 95%) and very high (>95%) based on the proportion of patients who always had access [18].

**Overall access:** was measured as the percentage or proportion of prescribed anti-hypertensive drugs available from either the hospital or the community pharmacy.

**Ethical approval:** ethical approval was obtained from the institutional review board of the Korle Bu Teaching Hospital (approval number: KBTH-IRB/000109/2018). Consent was sought from all patients before they were recruited in the study. Patient confidentiality was ensured.

## Results

**Socio-demographic characteristics of respondents:** out of the total of 310 questionnaires administered, 304(98%) of them were retrieved and were complete. Two hundred and thirty (76.2%) were females and were between the age group of 56 and 80 years. Almost all of them were subscribers of the National Health Insurance Scheme (NHIS). Two hundred and thirty-four respondents (77%) obtained their anti-hypertensive drugs by using NHIS card only and 59 respondents (19.4%) made some out-of-pocket payments for these medicines. Only 1 patient (0.3%) purchased anti-hypertensive with private insurance (Table 2).

**Table 2:** socio-demographic characteristics of study participants (N=310) recruited at the Korle Bu teaching hospital polyclinic (Ghana) from January to February 2019

Characteristic	n	%
**Age (years)**		
18-35	7	2.3
36-55	87	28.6
56-80	198	65.2
>80	12	3.9
**Gender**		
Male	74	23.8
Female	230	76.2
**Highest educational level**		
No formal education	73	23.5
Basic	118	38.9
Secondary	83	27.6
Tertiary	30	10.0
**Health Insurance subscriber (NHIS)**		
Yes	301	99.0
No	3	1.0
**Alcohol intake**		
Yes	50	16.5
No	254	83.5
**Payment method**		
Out-of-pocket	10	3.3
NHIS only	234	77
Private health insurance	1	0.3
Out-of-pocket + NHIS	59	19.4

**Clinical characteristics of respondents:** one hundred and ninety patients (62.5%) had been living with hypertension for more than 5 years. Ninety-six respondents (31.6%) had diabetes. Respondents who were prescribed two BP lowering drugs were 160 (52.6%) and 57 (18.8%) had prescription for only one BP lowering drug. Prescription containing four BP lowering drugs was issued to 15 respondents (4.9%). One hundred and five respondents (36%) were taking a statin, 162 (54.7%) were taking at least one drug for another chronic condition. One hundred and twenty-seven respondents (41.8%) had their BP controlled (Table 3).

**Table 3:** clinical characteristics of study participants (N=310) recruited at the Korle Bu teaching Hospital polyclinic (Ghana) from January to February 2019

Characteristic	n	%
**Comorbidity**		
With Diabetes	96	31.6
Without Diabetes	208	68.4
**Number of Years with hypertension**		
2 - 6 months	16	5.3
7 months-2 years	31	10.2
2 - 5 years	67	22.0
>5 years	190	62.5
**Herbal medication use**		
Yes	67	22.0
No	237	78.0
**Number of prescribed antihypertensive**		
1	57	18.8
2	160	52.6
3	71	23.4
4	15	4.9
5	1	0.3
**BP Control**		
Uncontrolled	177	58.2
Controlled	127	41.8
**Taking statin**		
No	187	64.0
Yes	105	36.0
**Taking aspirin**		
No	276	94.5
Yes	16	5.5
**Number of other chronic drugs**		
0	134	45.3
1	83	28.0
2	52	17.6
3	25	8.4
4	2	0.7

**Anti-hypertensive prescribed:** the five most frequently prescribed antihypertensive drugs were calcium channel blockers (n=264, 42.4%) and diuretics (n=39, 22.3%), angiotensin II receptor blockers (n=101, 16.2%), angiotensin converting enzyme inhibitors (n=82, 13.2%) and beta blockers (n=36, 5.8%) (Table 4).

**Table 4:** antihypertensive drug classes prescribed for study participants (N=310) recruited at the Korle Bu teaching hospital polyclinic (Ghana) from January to February 2019

Drug class	n (%)
Calcium channel blockers	264 (42.4%)
Diuretics	139 (22.3%)
Angiotensin II receptor blockers	101 (16.2)
Angiotensin converting enzyme inhibitor	82 (13.2%)
Beta blockers	36 (5.8%)

**Figure 1:**
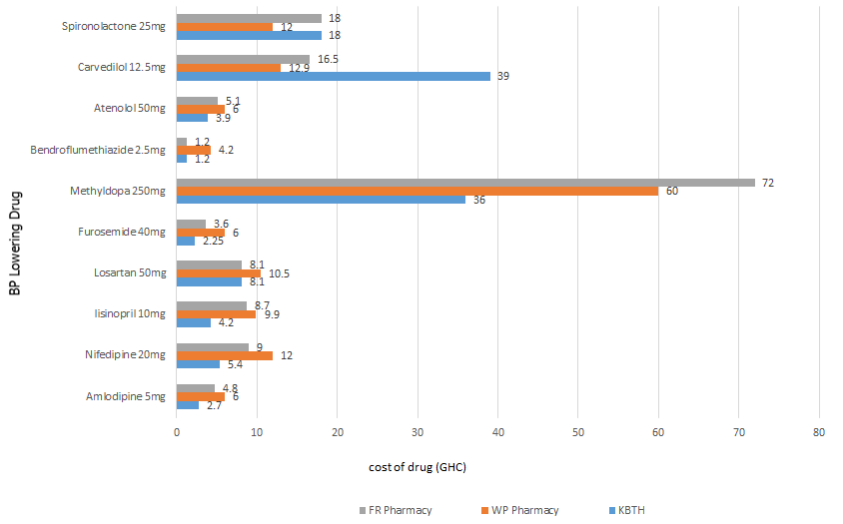
monthly cost of BP lowering medicines (N=10) surveyed at the Korle Bu teaching hospital polyclinic and community pharmacies (Ghana) from January to February 2019

**Cost of standard dose and out-of-pocket capacity-to-pay (CTP) for anti-hypertensive medicines:** the average cost of one-month supply of a standard dose of one anti-hypertensive medicine estimated from median prices was USD 1 at the hospital and USD 1.94 at the private facilities. The most expensive medicine was methyldopa (USD 7.5) at the hospital; (USD 13.8) at private facility (Figure 1). The anti-hypertensive with the least cost for one-month supply was Bendroflumethiazide (USD 0.25 at the hospital; USD 0.56 at private facility). The cost of 1-month supply of 4 BP lowering medicines was USD 4 at the hospital and USD 7.75) at the private pharmacy. The average capacity-to-pay out-of-pocket for a monthly supply of 4 anti-hypertensive medicines was US$ 66.24. About 13% (n=39) of patients had zero capacity to make any out-of-pocket payments for medicines. Less than 20% of patients had CTP < USD 21. Two hundred and sixty-two (86.2%) respondents could afford one BP lowering medicine out-of-pocket at the hospital whiles 249(81.9%) respondents could afford at the private pharmacy. Respondents who had affordability for two BP drugs at hospital were 249(81.9%) and at the private pharmacy it was 227(74.7%). Two hundred and thirty-four (77%) and 246 (80.9%) respondents could afford three drugs at hospital and private pharmacy respectively. At the hospital 74.7% had affordability for four drugs whiles 59.5% of patients could afford 4 drugs at the private pharmacy.

**Comparing affordability with blood pressure control:** out-of-pocket affordability at the hospital was positively correlated with blood pressure control (R=0.12, p=0.037). Out-of-pocket affordability at the private pharmacy was positively correlated with blood pressure control (R=0.12, p=0.037). In a logistic regression analysis, while controlling for demographics and clinical factors, affordability was more likely to result in blood pressure control, and this association was statistically significant (OR, 1.917; 95%CI: 1.013-3.630). Paying for anti-hypertensive medicines with NHIS was more likely to result in BP control than using out of-of-pocket only or NHIS plus out-of-pocket payment (OR, 2.185; 95% CI 1.215 - 3.927).

**Accessibility and availability of anti-hypertensive medicines:** only 45(15%) of respondent always obtained all their prescribed anti-hypertensive medication (continuous access) from the hospital pharmacy. Two hundred and forty five respondents (85%) obtained some of their anti-hypertensive medication from the hospital and had to procure some from another source, usually a private pharmacy. At the time of the study 60% of the anti-hypertensive medicines were available at the polyclinic pharmacy. Lisinopril, Atenolol, Carvedilol and spironolactone were not available. The mean overall access was 74.9% (SD ± 41.3). Patients access to anti-hypertensive medication at the hospital was more likely to result in BP control than otherwise (OR, 1.642; 95% C.I, 0.843-3.201) Respondents with controlled BP had a higher overall access or medicine acquisition (76.95%) than those who had uncontrolled BP (73.47%) although this was not statistically significant (p=0.471).

## Discussion

In this study, most patients obtained medication free with national health insurance, but some of them had affordability challenges because they needed to purchase some of their medication out-of-pocket at the private pharmacy. Accessibility was a challenge at the hospital. Patients who had affordability with health insurance at the hospital, and those with out-of-pocket affordability at the private pharmacy were more likely to have controlled BP than those who did not have affordability with health insurance and out-of-pocket affordability respectively. Study participants who had access were also more likely to have controlled BP than those who did not have access. The demographic characteristics of patients in this study are comparable to those of hypertensive patients in other lower middle-income countries [9]. A high proportion of our study participants were females probably because males are less likely to utilize health-care services than females [19]. However, in Ghana high prevalence of hypertension has been reported among men and women in both rural and urban settings [20,21]. In this study the prevalence was high among adults aged 55 years and above. Older patients have a higher risk of developing hypertension. Although a high proportion of participants were registered with the National Health Insurance Scheme (NHIS) some were still making out-of-pocket payments for anti-hypertensive medicines. This has also been shown by a recent study in Ghana, suggesting that the financial barrier to accessing anti-hypertensive medicines is still a challenge to some NHIS subscribers; due to out-of-pocket payments [14].

We found a significant proportion of patients living with chronic co-morbidities, and diabetes was the most common. Many hypertensive patients have co-morbid diabetes [22], which increases their cardiovascular risk. This co-existence is expected because diabetes is a risk factor for hypertension and hypertension has been reported as a risk factor for diabetes [23]. Many hypertensive diabetic patients require multiple medication to control their disease and reduce cardiovascular risk. As a result, affordability may be challenging for these patients. The majority of patients in our study were taking at least one drug for a chronic condition, and a significant proportion were taking a statin. While statins are essential in many hypertensive patients due to their value in cardiovascular risk reduction, affordability is likely to be lower for patients on statins, especially if they have to make some out-of-pocket payments [9].

Majority of participants were taking two or more anti-hypertensive medicines. Many patients with hypertension require two or more medicines to control blood pressure [8]. The long-standing hypertension (more than five years) which was found in majority of our patients may account for the use of multiple medication by study participants. In the management of hypertension, a step-care treatment is often recommended, but high risk patients may receive initial combination treatment [24,25]. Blood pressure control levels were low among study participants. This is consistent with the outcome of other studies [5,6]. Most patients were on calcium channel blockers and thiazide diuretics. This finding is consistent with guideline recommendations for this population [26,27].

The average cost of one month supply of one BP drug at the hospital was about half of the cost at the private pharmacy. This trend is consistent with other studies. [10,28] This situation could lead to reduced access of antihypertensive drugs if they are not available at the hospital. The average cost of the lowest priced generic BP lowering medicine for one month widely varied across medicines, reflecting the need to consider cost in drug selection. Although NHIS subscribed patients can access NHIS drug supply free of charge, participants could not obtain spironolactone supply for free because the cost of spironolactone was higher priced at the hospital than the price reimbursed by the NHIS. Carvedilol is however, not on the NHIS medicines list and therefore will not be reimbursed by the scheme. This suggests that while most antihypertensive drugs are free for NHIS patients, the reality of out-of-pocket payments exist for these patients prescribed Carvedilol.

The average capacity to pay in this study was lower than the average amount found in lower-middle income countries, but comparable to low-income countries [9]. More than a tenth of the participants had zero capacity to make any out-of-pocket payments for medicines. This suggests that these participants may have to borrow or not be able to acquire their medication if they have to pay out-of-pocket. Catastrophic health expenditure which leads to poverty among some African patients who make out-of-pocket payments has been reported in literature [29]. In one study, up to twenty percent of households were impoverished by catastrophic health spending [29]. The proportion of participants with affordability for two BP drugs at the hospital is lower than findings from lower-middle income countries which indicates that blood pressure lowering medicines are often not affordable [9]. Affordability of participants was also lower when they had to purchase at the private pharmacy and this is consistent with existing evidence [10,28]. A quarter of patients could not afford the medicines out-of-pocket from the hospital. This significant lack of affordability suggests that participants recruited for this study probably belong to a predominantly urban-poor population. Hypertensive patients often require other cardiovascular medication such as anti-platelets and statins. For our study participants who need these drugs, their affordability will be lowered further. Participants enrolled on the NHIS, and who obtained all their BP drugs were more likely to have controlled blood pressure than those who made some out-of-pocket payment. Other authors have also shown that one´s health insurance status is associated with BP control [30,31]. This finding further reinforces the need for policy makers to strengthen the NHIS as it is also likely to influence BP control, apart from reducing out-of-pocket payments and catastrophic health expenditures. Affordability for BP medication was more likely to result in BP control. A study by Attaei *et al*. has shown a similar relationship between affordability and BP control [9].

Availability of the lowest priced generic BP lowering medicines surveyed at the hospital was just above average but lower than the WHO target. This finding is consistent with recent evidence which shows that the availability of generic cardiovascular medicines in the public sector is low in lower middle-income countries [9,10]. This finding may reflect gaps in the public sector supply chain. In settings where national health insurance schemes provide medicines for non-communicable diseases (NCDs) free, availability is often low [32]. According to the World Health Organisation, access to medicines must be continuous. However, majority of our study participants did not have continuous access. Attaei *et al*. have shown in their study that a large proportion of hypertensive patients in low-income and middle-income countries do not have access to BP lowering medicines [9]. Low access at the hospital may mean that patients will often access medicines from private pharmacies where prices are higher, and NHIS subscribers are very likely to pay out-of-pocket for their BP drugs. Patients who always had access to anti-hypertensive drugs at the hospital were more likely to have controlled blood pressure than those who did not. This association was not statistically significant. However, the PURE study showed with statistical significance that patients who have access to BP lowering medicines are more likely to have controlled blood pressure [10]. The affordability and accessibility findings of this study show a gap in achieving the NCDs target on access at the hospital, and this is likely to be a limitation to cardiovascular disease mortality reduction among our study participants.

**Limitations of the study:** participants in the study were sampled from patients attending the polyclinic. Future studies should include hypertensive patients from other centers to enable generalization of the findings. This study did not account for expenditure on housing, transportation and utility in determining capacity to pay. Thus, affordability may be over-estimated.

## Conclusion

There were access challenges at the hospital although most patients obtained BP medication free. Out-of-pocket affordability is a challenge for some hypertensive patients. Strengthening the NHIS can improve access, and access to affordable anti-hypertensive medication can improve BP control among patients. Urgent attention to tackling access challenges at the hospital is needed. These findings provide an impetus for urgently evaluating access to affordable anti-hypertensive medicines in other hospitals in Ghana.

### What is known about this topic

- Where medicines are supplied free, access may be a challenge. In communities where medicines are accessible at health facilities, they may not be affordable.

### What this study adds

- This study adds to existing evidence on the positive relationship between access to affordable ant-hypertensive medicines and blood pressure control. It provides evidence on the potential influence of social health insurance use on hypertension control. It also provides evidence for developing facility-contextualized health policy interventions.
